# Supplementary material for: The Cultivar Effect on the Taste and Aroma Substances of Hakka Stir-Fried Green Tea from Guangdong
Source: Foods. 2023 May 20;12(10):2067. doi: 10.3390/foods12102067 (PMC10217579; doi:10.3390/foods12102067)
Supplement: Supplementary file 1 [file foods-12-02067-s001.zip › Figure S1.pdf]

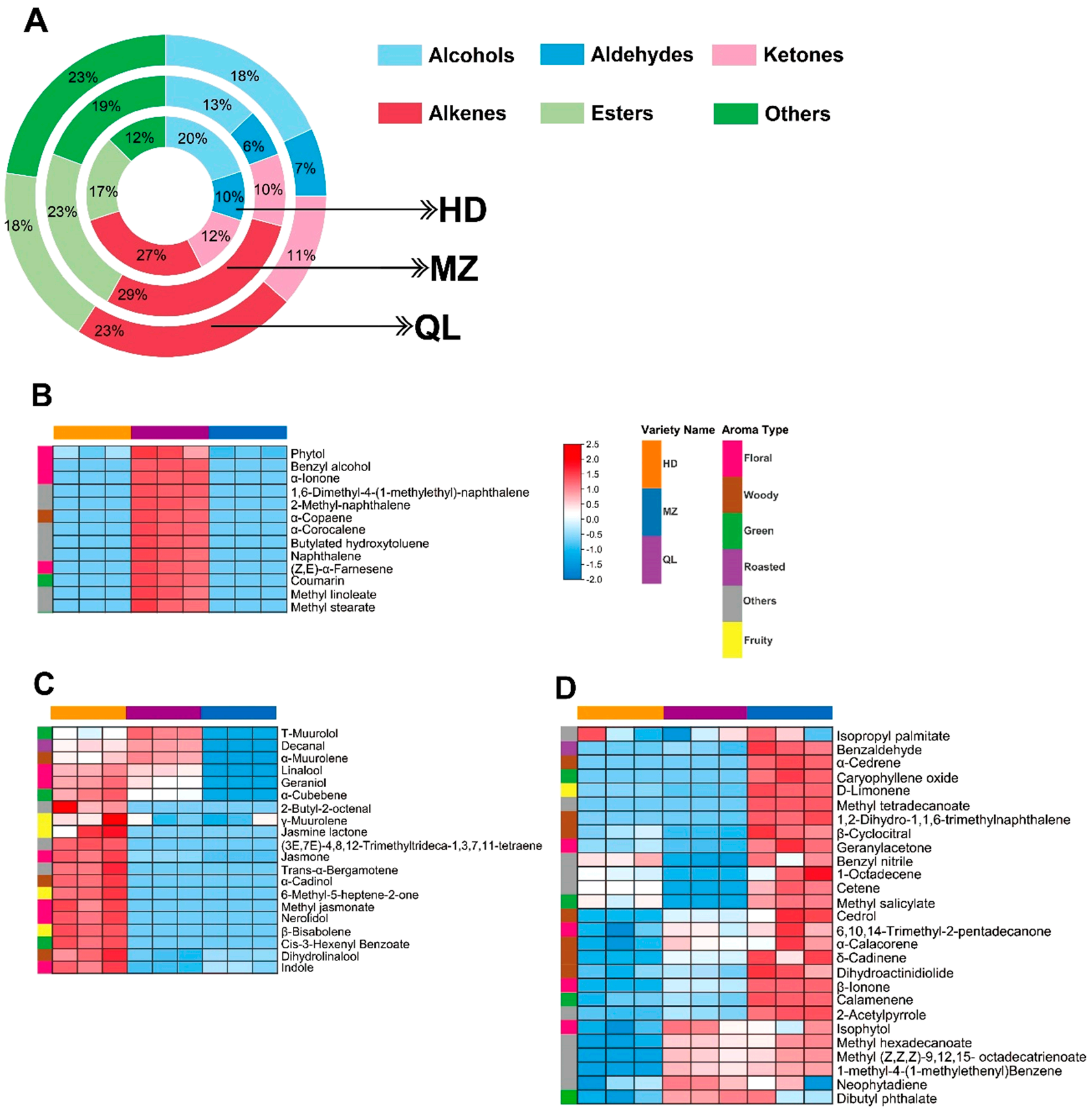

Figure S1. (A) Ratios of the 60 volatile compounds of HSGT and heatmaps of the major up-regulated volatile compounds in HSGTs made from (B) QL, (C) HD, and (D) MZ.
